# Innovative Approaches Using Lichen Enriched Media to Improve Isolation and Culturability of Lichen Associated Bacteria

**DOI:** 10.1371/journal.pone.0160328

**Published:** 2016-08-05

**Authors:** Elena G. Biosca, Raquel Flores, Ricardo D. Santander, José Luis Díez-Gil, Eva Barreno

**Affiliations:** 1 Departamento de Microbiología y Ecología, Facultad de Ciencias Biológicas, Universitat de València, Avda. Doctor Moliner 50, 46100, Burjassot, Valencia, Spain; 2 Departamento de Botánica e ICBIBE (Instituto Cavanilles de Biodiversidad y Biología Evolutiva), Facultad de Ciencias Biológicas, Universitat de València, Avda. Doctor Moliner 50, 46100, Burjassot, Valencia, Spain; 3 Grupo RETRACAR, Instituto de Investigación Sanitaria La Fe, Hospital Universitario y Politécnico La Fe, Avenida Fernando Abril Martorell, 106, 46026, Valencia, Spain; US Geological Survey, UNITED STATES

## Abstract

Lichens, self-supporting mutualistic associations between a fungal partner and one or more photosynthetic partners, also harbor non-photosynthetic bacteria. The diversity and contribution of these bacteria to the functioning of lichen symbiosis have recently begun to be studied, often by culture-independent techniques due to difficulties in their isolation and culture. However, culturing as yet unculturable lichenic bacteria is critical to unravel their potential functional roles in lichen symbiogenesis, to explore and exploit their biotechnological potential and for the description of new taxa. Our objective was to improve the recovery of lichen associated bacteria by developing novel isolation and culture approaches, initially using the lichen *Pseudevernia furfuracea*. We evaluated the effect of newly developed media enriched with novel lichen extracts, as well as the influence of thalli washing time and different disinfection and processing protocols of thalli. The developed methodology included: i) the use of lichen enriched media to mimic lichen nutrients, supplemented with the fungicide natamycin; ii) an extended washing of thalli to increase the recovery of ectolichenic bacteria, thus allowing the disinfection of thalli to be discarded, hence enhancing endolichenic bacteria recovery; and iii) the use of an antioxidant buffer to prevent or reduce oxidative stress during thalli disruption. The optimized methodology allowed significant increases in the number and diversity of culturable bacteria associated with *P*. *furfuracea*, and it was also successfully applied to the lichens *Ramalina farinacea* and *Parmotrema pseudotinctorum*. Furthermore, we provide, for the first time, data on the abundance of culturable ecto- and endolichenic bacteria that naturally colonize *P*. *furfuracea*, *R*. *farinacea* and *P*. *pseudotinctorum*, some of which were only able to grow on lichen enriched media. This innovative methodology is also applicable to other microorganisms inhabiting these and other lichen species.

## Introduction

Lichens are symbiotic systems that arise as self-supporting mutualistic associations between a fungal partner and one or more photosynthetic partners (unicellular green algae and/or cyanobacteria) [1,2]. They are unique phenotypes (holobionts) in which complex interactions enable the emergence of new structural and functional characteristics representing evolutionary innovations [3–5]. These novel properties allow the colonization of diverse habitats and survival under extreme environmental and climatic conditions [6–9], even in outer space [10]. In addition, the intrathalline coexistence of different algal lineages with diverse physiological patterns of abiotic stress tolerance could be advantageous to lichens [11–13].

These singular symbiotic associations also harbor other microorganisms such as non-photosynthetic bacteria, which are increasingly considered as integral components of the lichen thallus [6,7,14]. The presence of such bacteria within lichen thalli have long been known (reviewed in [8,9]), but recent studies, often by culture-independent methods, have begun to reveal their high diversity and abundance as well as some possible roles in lichen symbiosis [6,7,15–25]. A recent study using omic techniques has reported more than 800 bacterial species associated with the lichen *Lobaria pulmonaria* [26], revealing lichens to be a significant source of novel microorganisms. This same study also proposed the potential contribution of *L*. *pulmonaria* associated bacteria to various aspects related to symbiotic sustainability. In fact, bacterial communities are now recognized as stable, specific and structurally integrated partners in lichen multispecies symbiosis [8,27].

To date, only a few studies have reported the isolation of heterotrophic bacteria (requiring organic carbon for growth) from a reduced number of lichen species due to difficulties in isolating and culturing them [15,20,23,24,28–30]. This may be due to the methodologies currently used for the bacteriological analysis of lichens, as well as the use of conventional culture media that do not reproduce the complex nutritive conditions of lichen thalli [6,15,16,20,23,24,29,31]. Some heterotrophic bacteria associated with lichens may have special nutritional requirements not provided by standard culture media, hence they cannot grow on these media. However, culturing as yet unculturable bacterial partners is critical in order to unravel their multiple potential roles and interactions in lichen symbiogenesis, to explore and exploit their biotechnological potential, and also for the description of new bacterial taxa. Therefore, there is a need for innovative strategies that increase the recovery and improve the culturability of lichen associated bacteria.

The objective of this study was to develop a standardized methodology to improve the recovery of bacteria associated with lichens by using novel isolation and culture approaches. Since lichens are externally and internally colonized by bacterial communities [6,16,17,32,33], one purpose of this study was to elaborate isolation protocols to increase the recovery of ectolichenic (inhabiting external lichen surfaces) and endolichenic (inhabiting inner lichen tissues) culturable bacterial populations associated with lichens. One successful approach to improve the culturability of not-yet-cultured bacteria from environmental samples has been to modify culture media to mimic the natural environment [34]. Then, a second purpose was to obtain novel lichen extracts to develop growth media that mimics lichen nutrients. Additionally, the use of natamycin as a fungicide and the effect of different disinfection treatments and processing protocols were evaluated, to optimize the recovery of ectolichenic and endolichenic bacteria.

We initially used the lichen *Pseudevernia furfuracea* (L.) Zopf, belonging to the family *Parmeliaceae*. This lichen species was selected because of its wide distribution in temperate and cold regions in Europe and Asia, North Africa and Central-South America [35], as well as its presence in Mediterranean mountains. Thereafter, the innovative strategies developed were also evaluated in other lichen species such as *Ramalina farinacea* (L.) Ach. and *Parmotrema pseudotinctorum* (des Abb.) Hale.

## Materials and Methods

### Lichen samples, field sampling location and sampling procedure

**Fig 1:**
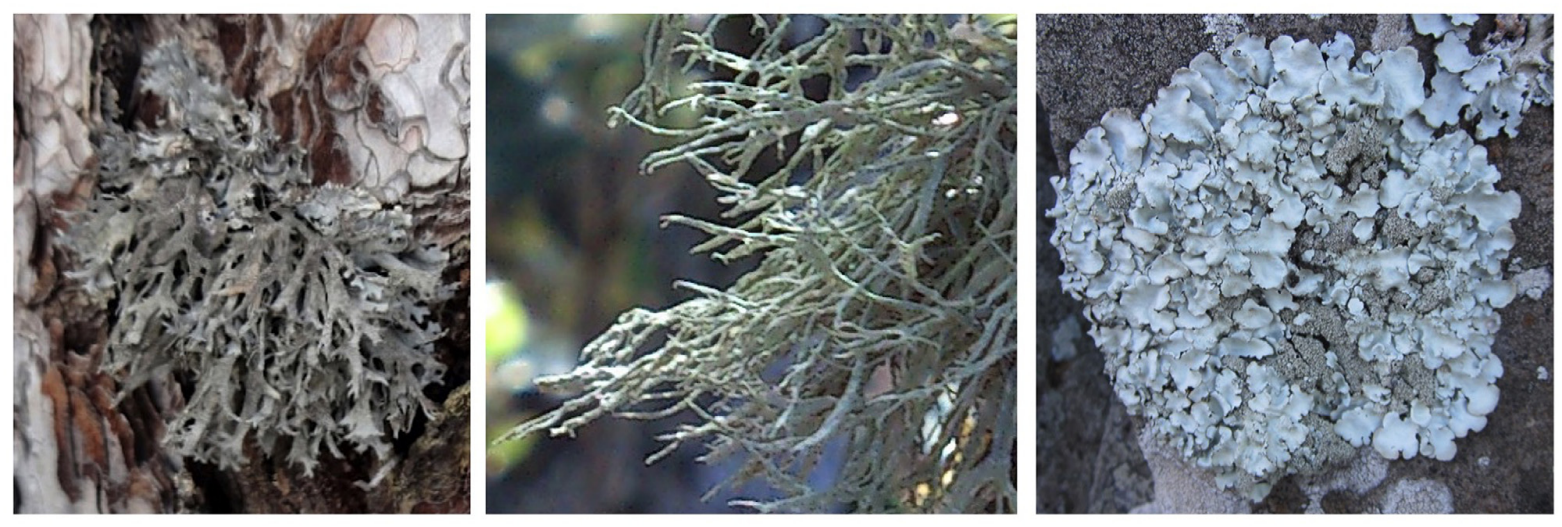
The three lichens species analyzed. The fruticose lichens *Pseudevernia furfuracea* (L.) Zopf (left) and *Ramalina farinacea* (L.) Ach. (centre) on *Pinus sylvestris* L. from the Javalambre mountains (Teruel, Spain) and *Pinus canariensis* Chr. Sm. ex DC. from La Esperanza mountain, Pista de Ovejeros (Tenerife, Canary Islands, Spain) respectively, and the foliose lichen *Parmotrema pseudotinctorum* (des Abb.) Hale (right) on volcanic rocks from El Palmar (Tenerife, Canary Islands, Spain).

Thalli of *P*. *furfuracea* (Fig 1, left) were collected from a *Pinus sylvestris* L. forest in a non-polluted area in the Javalambre mountains (40° 09′ 52.35″N, 1° 00′ 48.12″W), in Teruel (South Aragón, Spain). Thalli of *R*. *farinacea* (Fig 1, centre) and *P*. *pseudotinctorum* (Fig 1, right) were sampled from a *Pinus canariensis* Chr. Sm. ex DC. forest at La Esperanza mountain, Pista de Ovejeros, (28° 21′ 27,189″N, 16° 23′ 10.550″W) and volcanic rocks from El Palmar (28° 21′ 7.251″N, 16° 50′ 39.613″W), respectively, both located in Tenerife (Canary Islands, Spain). No specific permissions were required for sampling at the specified locations because these lichens are not considered protected species in Spain. Lichen samples were collected at each location from at least five arbitrarily selected trees or rocks within an area of about 50 m^2^, in several samplings in the years 2012 to 2014. On each sampling, at least 10 lichen thalli were collected from each location to analyze single or bulk lichen thalli samples. Part of these thalli were also used to obtain fresh lichen extracts to prepare lichen enriched media (see below). Specimens appearing healthy were carefully removed from tree bark or rocks using sterile gloves and transferred into sterile plastic Petri plates stored in individual plastic bags. Thalli samples were transported and stored under refrigeration until processing within 24 h after sampling.

### Initial protocol for isolation of lichen associated bacteria

Since there is no standardized methodology for the bacteriological analysis of lichen associated bacteria, initially a protocol based on Cardinale et al. [15], Grube et al. [6] and Selbmann et al. [20] was used for the isolation of bacteria associated with two *P*. *furfuracea* single thallus samples. Briefly, each single thallus was washed with sterile distilled water for 1 min, and 0.2 g subsamples were cut using sterile scalpels and placed in sterile Petri dishes. These subsamples were surface disinfected with ethanol 70% (vol:vol) for 1 min, washed twice for 5 min in sterile distilled water and aseptically comminuted (cut into very small particles using sterile scapels) in sterile saline (0.9% NaCl in distilled water, pH 7.0) solution (SS) in sterile Petri dishes. Then, comminuted samples were tenfold serially diluted in sterile 10 mM phosphate buffered saline (PBS), pH 7.0, and spread plated on the complex non selective medium King’s B (KB) [36], routinely used for the isolation of plant associated bacteria. To prevent fungal growth, this medium was supplemented with filtered sterilized cycloheximide (at a final concentration of 50 mg/L), a widely used fungicide for the analysis of environmental and plant samples [37–39]. Plates were incubated for a week at 25°C under dark conditions, and bacterial colonies counted to determine colony forming units per gram (CFU/g) of thallus tissue. Morphologically different colonies were purified on the same medium and cryopreserved at -80°C in 25% (vol:vol) glycerol.

### Modification of protocol of bacterial isolation to improve the recovery of bacteria associated with *P*. *furfuracea*

To improve the recovery of lichen associated bacteria two modifications of the initial protocol for the isolation of bacteria were assayed by analyzing three single thallus samples in independent experiments.

Firstly, since lichens are externally colonized by bacterial communities forming biofilms [6,16,17,32,33], to increase the isolation of ectolichenic bacteria three different thallus samples were washed for 90 min in 250 ml flasks with 50 ml of sterile Ringer's solution [40] containing 0.05% of the surfactant Tween 20 (RST) in an orbital shaker (200 r.p.m) at room temperature, after being briefly washed (1 min) with sterile distilled water to remove environmental powder. To quantify the recovered bacteria from lichen surfaces, aliquots were taken at different periods (5, 30, 60 and 90 min) during thalli washing, serially tenfold diluted in PBS and plated on KB agar.

Secondly, to improve the isolation of endolichenic bacteria and prevent, or reduce, the oxidative stress that may occur during thalli processing, which in turn could affect the isolation of lichenic bacteria, 0.2 g washed and surface disinfected thallus samples were aseptically comminuted in 3 ml of filter sterilized antioxidant maceration buffer (AMB) [41] instead of SS. Thallus samples processed in SS were used for comparison. Comminuted thallus samples were slightly shaken and incubated on ice for 5–10 min before plating.

For the isolation of ecto- and endolichenic bacteria, aliquots from thallus washings as well as disinfected and comminuted washed thallus suspensions, respectively, were serially tenfold diluted in PBS and plated (0.1 mL) in duplicate on KB and minimal media, all supplemented with cycloheximide. Since in earlier studies some lichenic bacteria, such as *Methylobacterium* sp., grew during *P*. *furfuracea* phycobiont isolation in Bold’s Basal Medium (BBM) minimal medium for algae growth [42], both BBM and Methanol Mineral Salts (MMS) medium [43], selective for *Methylobacterium*, were also included in these assays.

Inoculated plates were incubated for a week at 25°C under dark conditions and bacterial colonies were counted. Ecto- and endolichenic culturable bacteria populations were estimated by calculating mean values of CFU/g from thallus washings and disinfected and comminuted washed thallus, respectively. A random selection of colonies showing different morphologies on each medium were purified and cryopreserved, as mentioned above.

### Development of novel lichen enriched media

To further increase the recovery of *P*. *furfuracea* associated culturable bacteria, we tried to mimic the nutritional conditions of this lichen by developing several lichen enriched media adding *P*. *furfuracea* thalli extract to different minimal solid media (1.5% agar), supplemented, or not, with carbon sources. For this purpose, stock solutions with the different media compounds, including carbon sources and the novel lichen extract, were sterilized separately and then added to the appropriate volume of sterile melted agar in distilled water. We selected the minimal medium AB [44], which was supplemented, or not, with filtered sterilized 0.5% glucose (G) and/or 0.5% mannitol (M) (according to [15]) (AB, ABG, ABM and ABGM) and/or 0.5% fresh lichen extract (L) (ABL, ABLG, ABLM and ABLGM). For lichen enriched minimal media, 5% fresh lichen extract was prepared with *P*. *furfuracea* thalli that were rinsed for 1 min with sterile distilled water and then washed with sterile RST for 30 min at 200 r.p.m. at room temperature in an orbital shaker, triturated in a blender and filter sterilized (including the use of 0.8-μm pore size polycarbonate pre-filters). For larger amounts of lichen extract preparation an additional centrifugation step (6100 *x* g, Beckman Coulter JS-5.3 rotor) was included to ease pre-filtration and filter sterilization. BBM and MMS minimal culture media supplemented, or not, with lichen extracts were also used in some assays. All media were amended with cycloheximide as described above.

### Evaluation of lichen enriched media on the growth of *P*. *furfuracea* bacterial strains

To investigate the effect of the addition of lichen extract to minimal media on the growth of *P*. *furfuracea* bacterial strains, AB minimal medium (amended, or not, with glucose and/or mannitol and/or lichen extract) was selected to test the growth of a laboratory collection of 139 lichenic bacterial strains. Additionally, eight reference non-lichenic strains from the Spanish Type Culture Collection (CECT) (*Bacillus* cereus CECT 495, *Enterobacter cloacae* CECT 194, *Enterococcus faecalis* CECT 481, *Escherichia coli* CECT 101, *Micrococcus luteus* CECT 245, *Pseudomonas fluorescens* CECT 378, *Salmonella enterica* CECT 443 and *Serratia marcescens* CECT 159) were included for comparative purposes. Bacterial inocula were prepared from cultures grown on KB plates at 25°C for 48 h, washed and re-suspended in sterile PBS at a cell concentration of about 10^8^ CFU/ml. Each strain was spot inoculated with a multipoint inoculator (Denley Instruments Ltd, UK), at least in duplicate, on all lichen enriched and lichen free media, including KB medium as growth control, which was used for purification of the assayed strains. Growth results were recorded and classified either in two (0, no growth; 1, growth) or three categories (0, no growth; 1, growth on the inoculated spot; 2, abundant growth on the inoculated spot). Moreover, to determine the effect of incubation time on bacterial growth, plates were incubated for 96 h at 25°C and growth recorded after 24 h and 96 h.

### Application of the improved methodology for the isolation and culture of bacteria associated with *P*. *furfuracea* from thalli samples

To further evaluate the modified processing protocol (extended washing of thalli with RST and thalli disruption in AMB buffer) and the lichen enriched media developed in this study, new *P*. *furfuracea* thalli from additional samplings in the Javalambre mountains were analyzed, at first using single thallus and thereafter bulk thalli samples.

#### Single lichen thallus

Each sample of 0.2 g of *P*. *furfuracea* thallus was processed according to the methodology mentioned above, and ecto- and endolichenic culturable bacteria populations were estimated in duplicate on lichen enriched (ABL, ABLG, ABLGM) and lichen free media (AB, ABG, ABGM) as well as on KB, and incubated at 25°C for 3 days under dark conditions.

#### Bulk lichen thalli

Bulk samples of 1 g of *P*. *furfuracea* thalli (0.2 g sub-samples of five specimens from five different trees) from two different samplings in the same locality were analyzed. In this case, counts of ectolichenic and endolichenic culturable bacteria of *P*. *furfuracea* were determined in triplicate on high (ABLGM) and low nutrient (ABL) lichen enriched media, and also on lichen free ABGM and KB media. In addition, to favor the recovery of slow growing bacteria, the incubation period was extended up to 15 days (at 25°C under dark conditions), and the number of colonies appearing on plates was recorded at several day intervals. Furthermore, to initially estimate the influence of the lichen extract on the diversity of the recovered bacteria, the different colonial morphologies observed in the different culture media were recorded at the end of the experiment.

In both cases, colonies showing distinct phenotypes were randomly selected, purified mostly on KB plates and cryopreserved as described above.

### Optimization of isolation protocol and its application to improve the recovery of lichen associated bacteria

In order to optimize bacterial isolation from lichen thalli, additional modifications were assayed to reduce the growth of filamentous fungi and to further increase the recovery of lichenic bacteria.

#### Reduction of fungal growth on bacterial isolation plates

Since some filamentous fungi hindered the recovery of slow growing lichen associated bacteria, natamycin, described as a more efficient antifungal agent than cycloheximide and without health risks [39], was tested. The effect of both fungicides on a selection of seven different filamentous fungi often growing on bacterial isolation plates was compared on KB medium, where they grew more rapidly. The KB medium was amended with 21.6 mg/L natamycin [39] or 50 mg/L cycloheximide, using KB plates without fungicide as a control. Plates were incubated at 25°C under dark conditions, and the effect of the addition of each of these two fungicides on the recovery of lichenic bacteria was monitored over 7 days.

#### Evaluation of different thalli processing protocols to further improve the recovery of lichenic bacteria

Since our previous results seemed to indicate that the culturability of endolichenic bacteria could be affected by the disinfection step, which is performed after an extended washing of lichen thalli samples, we first assessed if this washing step was sufficient to recover most of the ectolichenic bacteria before isolating the endolichenic ones. Afterwards, we assayed the effect of different thalli disinfection protocols on these same washed thalli: *i)* 70% ethanol (vol:vol) for 30 and 60 sec; *ii)* 8% hydrogen peroxide (vol:vol) for 5 minutes, comparing them with respect to washed samples processed without undergoing any disinfection treatment.

At the same time, to try to speed up the isolation of bacteria from lichen samples, thalli disruption by comminution (taking about 5–10 minutes per sample) was compared with crushing (taking about 1.5 minutes per sample). In the second case, samples were crushed inside a sterile plastic bag with a net, using a rubber hammer [45]. In both cases, tissue disruption was performed in AMB. Aliquots of disrupted thalli extracts were serially tenfold diluted, plated on KB and ABGM in duplicate and incubated for one week at 25°C under dark conditions.

Once the optimal isolation and culture strategies for the recovery of lichenic bacteria were established, the innovative methodology developed was tested for the recovery of bacteria from additional thalli samples of *P*. *furfuracea* from Javalambre, as well as from the lichen species *R*. *farinacea* and *P*. *pseudotinctorum* from Tenerife.

For further studies, a random selection of colonies showing different morphotypes on isolation plates from all thalli analyzed were purified and cryopreserved.

### Statistical analysis

Data from culturable bacterial counts were expressed as the means of two or three determinations by each medium and bacteriological analysis. Data that did not follow a normal distribution were analyzed by the Mann-Whitney non-parametric U test, or normalized by logarithmic transformation. Statistical significances of the differences between two normalized means were analyzed using a parametric Student t test, and in the case of three or more means with an ANOVA followed by Bonferroni or Dunnet post-hoc analysis. Categorical variables (bacterial growth, incubation time and growth media) were compared using the Chi-square test. Multivariate analysis was performed using a logistic regression model to determine the effect of adding lichen extract to culture media, the incubation time and the origin (ecto- or endolichenic) of strains on the growth of a collection of *P*. *furfuracea* bacterial strains. A p value below 0.05 was considered significant. Data were analyzed with SPSS 19.0 software (IBM SPSS Statistics).

## Results and Discussion

The casual isolation of *Methylobacterium* sp. in BBM minimal media used for *P*. *furfuracea* phycobionts isolation was the beginning of an investigation to study the culturable bacteria associated with this lichen. Initial bacteriological analysis of *P*. *furfuracea* thallus samples using SS and complex nutrient rich KB medium yielded low bacterial counts (10^2^ CFU/g), similar to that described by Cardinale et al. [15] for different lichen species, including *P*. *furfuracea*. To improve the recovery of *P*. *furfuracea* associated bacteria and, since there is no standardized protocol, we developed and optimized a methodology of isolation and culture that was thereafter successfully applied to other lichen species.

### Extended thalli washing, antioxidant buffer and minimal media improve the recovery of *P*. *furfuracea* associated bacteria

Since bacterial communities colonize the lichen thallus surface in a biofilm-like manner [6,16,17,32,33], extended washing with a surfactant was used to improve the recovery of ectolichenic bacteria. This increase in the washing period of single thallus samples from 5 to 90 min, approximately doubled the number of recovered bacterial isolates on KB plates (from 1.4 x 10^2^ CFU/g after 5 min to 2.2, 2.7 and 3.3 x 10^2^ CFU/g after 30, 60 and 90 min, respectively).

Because the production of reactive oxygen species is stimulated in some lichens by wounding [46], and oxidative stress can inhibit bacterial growth [47,48], an antioxidant buffer, also used for the isolation of some plant associated bacteria [45,49], was utilized to prevent or reduce oxidative stress of endolichenic bacteria during thalli disruption. The isolation of endolichenic bacteria from the single thallus samples analyzed by comminution in AMB instead of SS, increased mean bacterial counts about one log unit (from 10^2^ to 10^3^ CFU/g) after a week of incubation on complex KB medium. In addition, bacterial colonies isolated with AMB appeared earlier on KB plates. Both results suggested some kind of oxidative stress during thalli disruption that can inhibit or reduce the growth of some lichenic bacteria.

**Fig 2:**
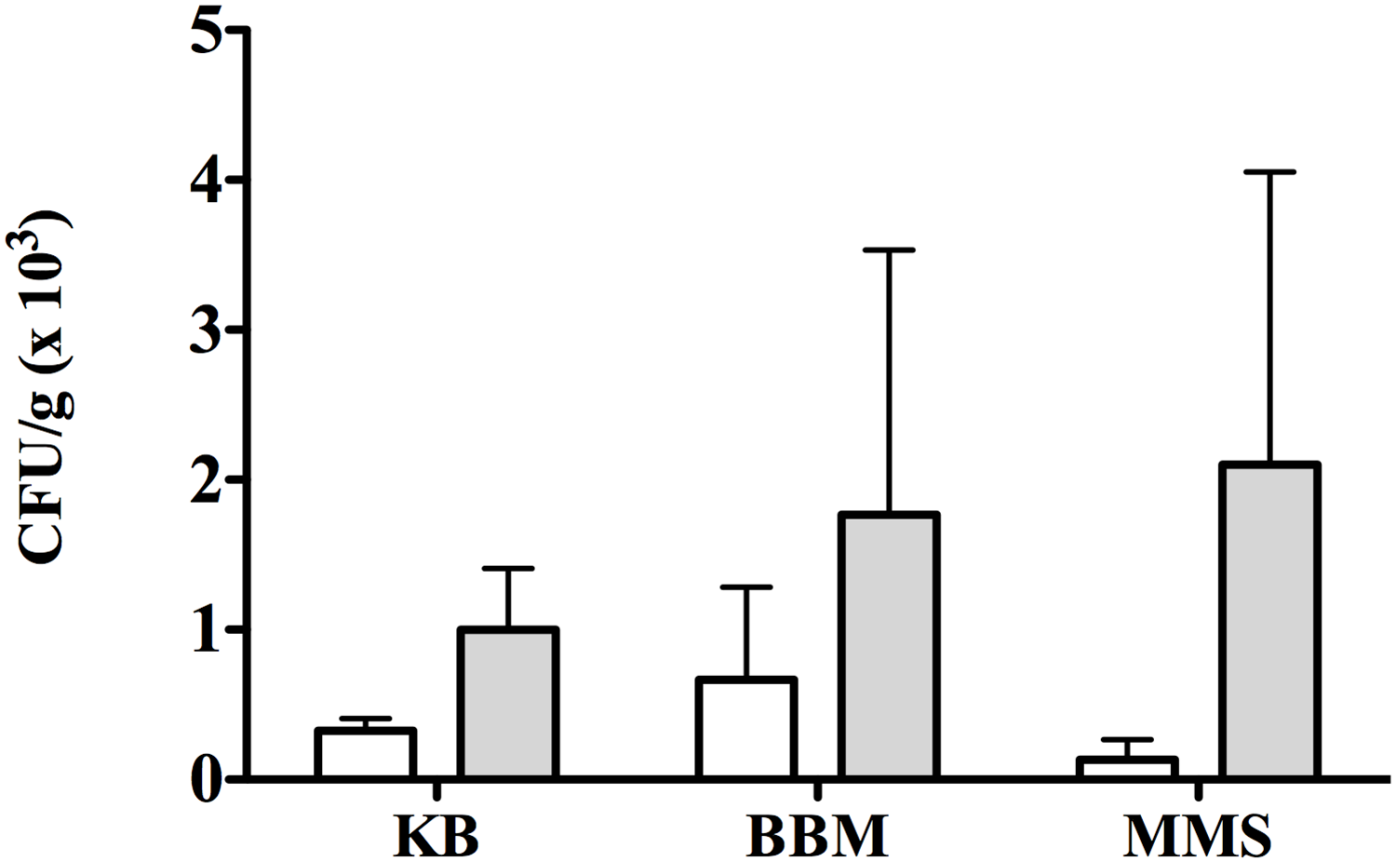
Improved recovery of *P*. *furfuracea* associated bacteria with a modified processing protocol and minimal media. Mean culturable counts (CFU/g) of *P*. *furfuracea* ectolichenic bacteria from washed thallus (white bars) and endolichenic bacteria from thallus tissue (grey bars) on complex medium KB and minimal media BBM and MMS, after 7 days of incubation at 25°C under dark conditions. Each bar represents the mean value of counts on each medium from three different thallus samples analyzed in independent experiments. Standard deviations are indicated by vertical lines. There were no significant (p>0.05) differences among culture media nor between ecto- and endolichenic bacteria.

Based on the above mentioned results, extended washing and the AMB antioxidant buffer were applied for the analysis of new single thallus samples to estimate ecto- and endolichenic bacterial populations. Different types of medium were used: complex rich medium (KB); the minimal medium BBM; and a selective minimal medium for *Methylobacterium* spp. (MMS) (Fig 2). After 7 days of incubation at 25°C ectolichenic mean culturable counts were lower than endolichenic ones although these differences were not statistically significant. Interestingly, mean counts of endolichenic bacteria on minimal media were higher than those on complex KB medium. Similarly, mean counts of ectolichenic bacteria were also higher on BBM than on KB plates, but not on MMS medium. Despite no significant differences being observed among KB, BBM and MMS (p>0.05), probably due to variability of counts obtained with different thalli, the obtained results could be attributed to a large number of oligotrophic bacteria in the lichen thallus which were unable to grow on the complex rich medium but, interestingly, able to grow on BBM where the only organic nutrients were thallus extracts co-inoculated with bacteria on isolation plates. These results lead us to hypothesize that the culturability of lichen associated bacteria could be improved by using minimal media enriched with lichen extracts.

### Lichen enriched media with novel lichen extracts improve the growth of a collection of *P*. *furfuracea* bacterial strains

**Fig 3:**
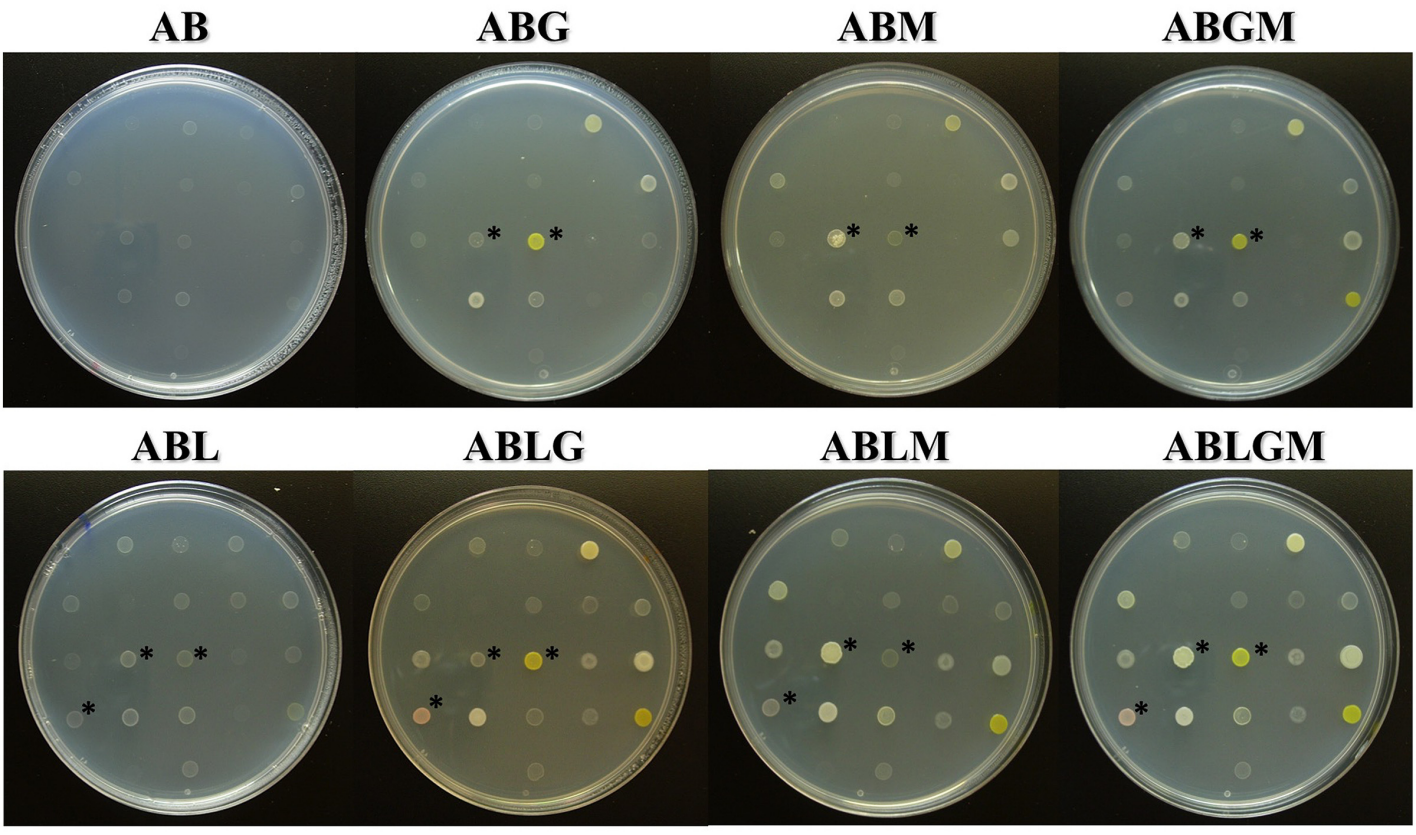
Comparison of the growth of *P*. *furfuracea* bacterial strains on lichen enriched *versus* lichen free minimal media (containing, or not, carbon sources). Representative pictures showing the growth of selected bacterial strains isolated from *P*. *furfuracea* on the minimal medium AB supplemented, or not, with lichen extract (L) and/or with carbon sources (glucose, G; mannitol, M; or glucose and mannitol, GM) after 96 h of incubation at 25°C under dark conditions. Some details of differential growth of some strains are indicated with black asterisks. All assayed strains grew on control medium KB in which they were purified.

To check our hypothesis, we decided to use a standard minimal medium (AB, containing, or not, carbon sources) enriched, or not, with the novel lichen extract to evaluate their effect on the growth of a collection of 139 bacterial strains previously isolated from *P*. *furfuracea*, as well some reference bacterial strains from non-lichenic sources. Representative pictures of the growth of *P*. *furfuracea* bacterial strains on AB, supplemented, or not, with lichen extract and/or carbon sources are shown in Fig 3. Greater growth on all lichen enriched media with carbon sources was observed for some bacterial strains when compared to lichen free media supplemented only with carbon sources. No growth was recorded on AB plates without carbon sources nor lichen extract. Furthermore, for some strains, growth was more abundant on ABLGM medium. In addition, the percentage of strains that grew on lichen enriched media (91.7%) was significantly higher (p<0.001) than the percentage which grew on the same media without lichen extract (66%) after 96 h of incubation.

**Fig 4:**
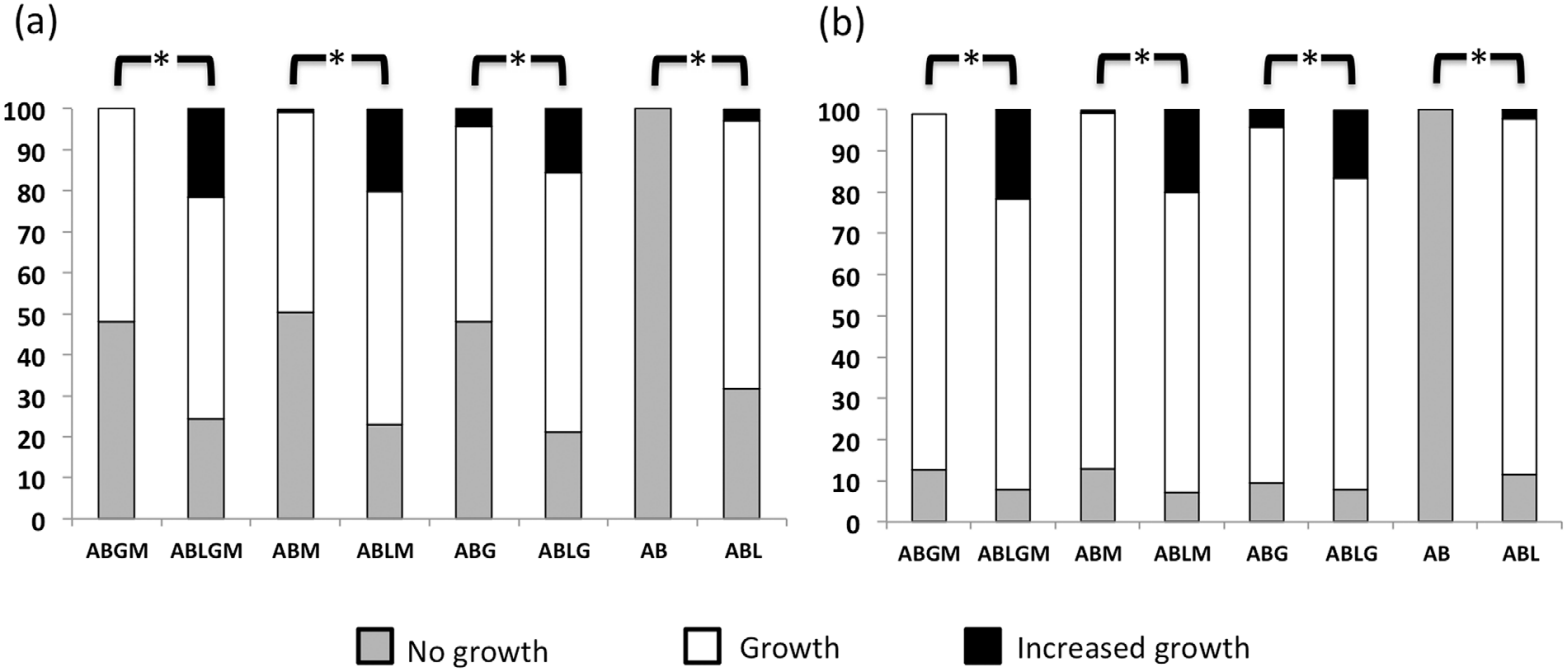
Effect of lichen enriched media and incubation time on the growth of *P*. *furfuracea* bacterial strains. The 100% stacked column chart of growth of 139 *P*. *furfuracea* bacterial strains on the minimal medium AB supplemented, or not, with lichen extract (L) and/or with carbon sources (glucose, G; mannitol, M; or glucose and mannitol, GM) after 24 h (a) and 96 h (b) of incubation at 25°C under dark conditions. Bacterial growth on each assayed medium was compared with control medium KB, which was arbitrarily set at 100% growth. Results were grouped in three categories (no growth, growth on the inoculated spot and abundant growth on the inoculated spot). Data are from one experiment performed by duplicate. Significant (p<0.001) differences between two culture media are indicated by asterisks above stacked columns. Significant (p<0.001) differences were also found between 24 h (a) and 96 h (b) of incubation.

The effect of incubation time on bacterial growth was also investigated after 24 h and 96 h of incubation. As shown in Fig 4, a larger percentage of strains grew on lichen enriched media than on lichen free media, after 96 h *versus* 24 h of incubation. Moreover, significant differences (p<0.001) were also found among the different lichen enriched media, with a larger percentage of strains showing abundant growth on ABLGM medium (20.4%), followed by ABLM (19.7%) and ABLG (16.4%) media, and a lower percentage on ABL medium (2.7%), after 96 h (Fig 4b). Interestingly, non-lichenic reference strains from CECT also grew in a higher percentage (78.1%) on lichen enriched than on lichen free (59%) media after 96 h of incubation. It is worth noting that most of the assayed strains were able to grow on both complex KB (control medium) and nutrient poor ABL media, suggesting that many of them are facultative oligotrophs, able to adapt to a wide range of nutrient concentrations, as reported for other bacteria from nutrient limited environments [50].

Furthermore, a logistic regression analysis demonstrated that the addition of lichen extract to the culture medium has the strongest effect on bacterial growth, regardless of the other variables studied. We found a fivefold greater likelihood of bacterial growth on media supplemented with lichen extract than on lichen free media (odds ratio = 5.2; 95% confidence interval = 4.2–6.4; p<0.001) (Table 1). As expected, the incubation time multiplied the likelihood of bacterial growth by 3 (odds ratio = 3.3; 95% confidence interval = 2.7–4.0; p<0.001) (Table 1), independently of the presence of lichen extract on the medium or the origin of the strains.

**Table 1:** Logistic regression model of bacterial growth probability.

Factors	β (SE)	Odds Ratio (95% CI)	P value
Medium with lichen extract			
Yes	1.64 (0.1)	5.18 (4.21–6.37)	< 0.001
No*			
Incubation time			
96 h	1.19 (0.1)	3.28 (2.68–4.01)	< 0.001
24 h*			
Strain origin			
Endolichenic	0.14 (0.12)	1.15 (0.91–1.4)	0.25
Ectolichenic*			
Constant	-1.69 (0.16)		

β (SE), β coefficient and its standard error (SE); CI, confidence interval.

* Reference category

Taken together, the former results demonstrated that media mimicking *P*. *furfuracea* thalli nutrients improve the growth of bacterial strains associated with this lichen, suggesting that lichen enriched media can be applied for the isolation of bacteria associated to this, and other lichen species.

### Lichen enriched media and extended incubation improve the recovery of culturable bacteria associated with *P*. *furfuracea*

**Fig 5:**
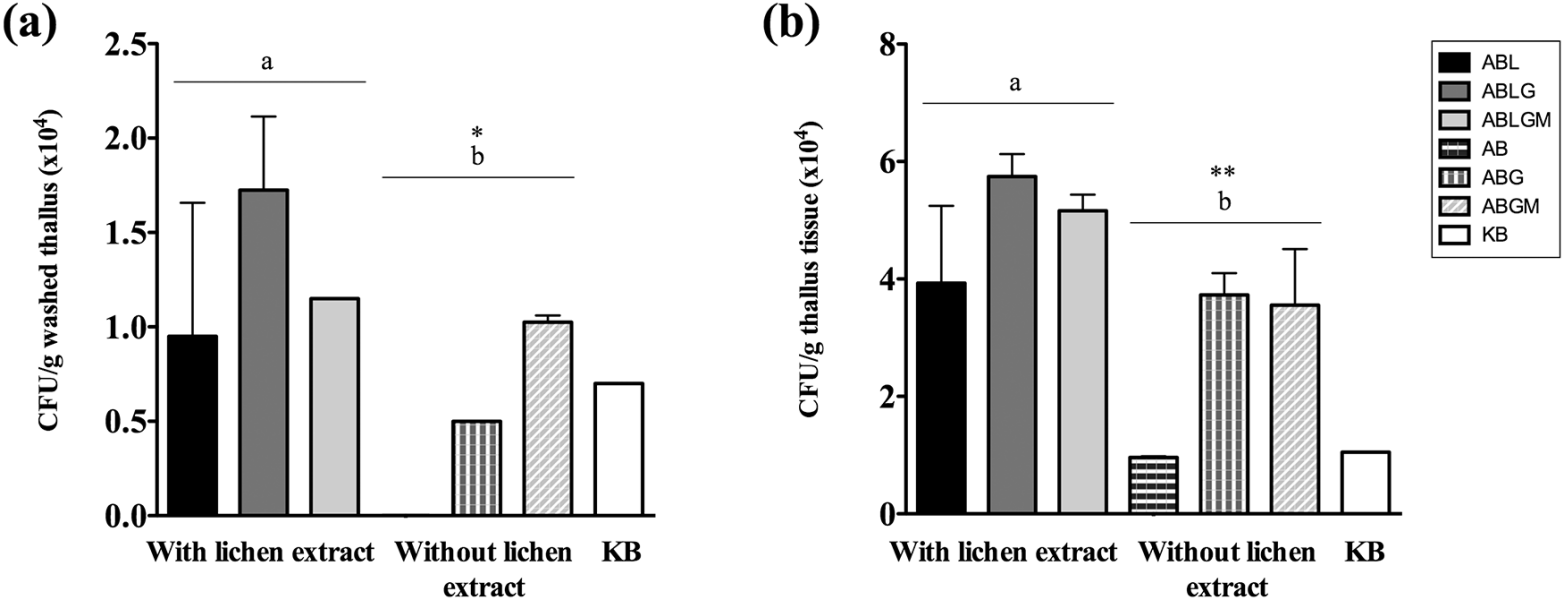
Effect of lichen enriched media on the recovery of *P*. *furfuracea* associated bacteria from thallus samples. Mean culturable counts (CFU/g) of *P*. *furfuracea* ectolichenic bacteria from washed thallus (a) and endolichenic bacteria from thallus tissue (b) on the minimal medium AB supplemented, or not, with lichen extracts (L) and/or with carbon sources (glucose, G or glucose and mannitol, GM) and KB medium, after 3 days of incubation at 25°C under dark conditions. Each bar represents the mean value of duplicate counts on each medium from thallus samples. Standard deviations are indicated by vertical lines. Significant differences between culture media supplemented, or not, with lichen extract are indicated by different letters shown above the error bars (* p<0.05; ** p<0.01).

The newly developed lichen enriched media and the modified processing protocol were preliminary assayed on a single thallus of *P*. *furfuracea*. With this new methodology, bacterial population sizes increased from 10^3^ CFU/g in initial experiments to 10^4^ CFU/g (Fig 5). After just 3 days of incubation at 25°C, ecto- and endolichenic bacteria from *P*. *furfuracea* thalli were recovered in higher numbers on all lichen enriched media, followed by lichen free media and/or KB (Fig 5). Moreover, bacterial counts on ABLG and ABLGM were higher than those obtained on ABL (Fig 5). A comparative analysis revealed significantly higher bacterial counts on lichen enriched minimal media than on lichen free or KB media (p<0.05) (Fig 5a and 5b). Although the nutritional thallus environment is mimicked by lichen enriched media it is worth noting that in nature bacteria are located in thallus interspaces where hyphal contents are limited within hyphae, and the interhyphal environment may differ from the macerated thallus used for lichen extract preparation, since some cellular contents may prevent bacterial growth (i.e. oxidative stress). However, our results demonstrate that a suitable isolation and culture methodology significantly improves the recovery of *P*. *furfuracea* associated bacteria, in part by contributing unique nutrients and/or unknown growth factors supplied by the lichen, which is difficult to provide with a synthetic medium. This is in accordance with previous studies, some of which were carried out by our group, showing that nutrients from the environment of interest can be included in media formulation, or directly used to stimulate bacterial growth or to recover non-culturable bacteria [51–58].

**Fig 6:**
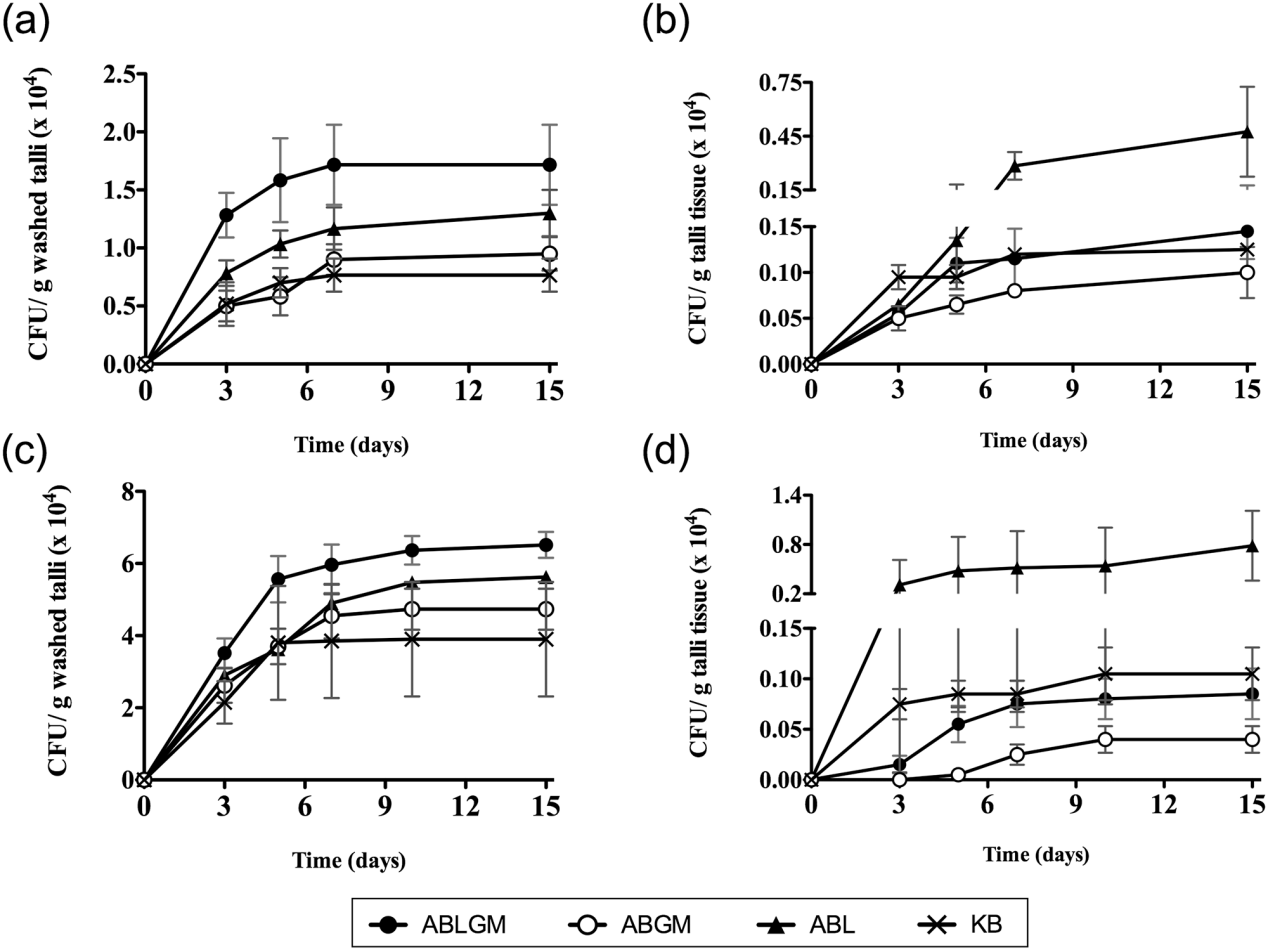
Effect of lichen enriched media and incubation time on the recovery of *P*. *furfuracea* associated bacteria from bulk thalli samples. Mean culturable counts (CFU/g) of *P*. *furfuracea* bacteria from two different samplings (a,b and c,d). Ectolichenic bacteria were estimated from washed thalli (a,c) and endolichenic bacteria from thalli tissue (b,d) of bulk samples (five thalli from different trees) on lichen enriched media with or without carbon sources (ABLGM and ABL), and also on lichen free medium with carbon sources (ABGM) and KB, with increasing incubation time up to 15 days at 25°C under dark conditions. Each data point represents the mean value of triplicate counts on each medium from the same bulk thalli sample. Standard deviations are indicated by vertical lines.

Since lichen thalli are considered as nutrient limited ecosystems [6] that can be colonized by facultative oligotrophic bacteria, we selected ABL and ABLGM media to estimate ecto- and endolichenic bacterial populations associated with *P*. *furfuracea*, using thalli bulk samples from two additional samplings, and including ABGM and KB media for comparison. As shown in Fig 6, mean bacterial counts on lichen enriched media ABLGM and ABL were higher than those obtained with lichen free ABGM and KB. Moreover, the number of colonial morphologies (indicative of bacterial diversity) was significantly higher on lichen enriched media than on lichen free media (p<0.05) (S1 Fig). Interestingly, the presence of carbon sources in minimal media had a favorable effect on the culturability of lichenic bacteria, but not on complex KB medium, which contains glycerol (1%) and also peptone (2%). Related to this, a growth inhibition of bacteria from nutrient poor environments has been previously reported by peptone (1%), casamino acids (1–2%) and yeast extract (0.1%) [59]. The former could also explain the low recovery of culturable lichenic bacteria reported on tryptone yeast extract medium [15,20], and probably on other complex media [24,29,31]. This should be taken into consideration for future studies. Furthermore, our present results, together with the fact that lichen samples were taken from a pine forest free of nitrogen contamination, lead us to hypothesize that the sensitivity of this lichen species to nitrogen pollution could be related to the sensitivity to nitrogen excess of some *P*. *furfuracea* associated bacteria, which is currently under investigation.

Because extended incubation periods increase the recovery of slow growing bacteria, particularly on low nutrient media [51,52,55,60], we also assessed the effect of incubation time on bacterial colony number stabilization. As shown in Fig 6, in general, mean bacterial counts increased rapidly within the first 3 to 5 days, especially on lichen enriched media, stabilizing in the following days. On complex KB medium, counts stabilized much earlier (about one week) compared to minimal media (about two weeks) (Fig 6). Accordingly, an incubation period of 15 days was selected for subsequent bacteriological studies.

The comparative analysis of ecto- and endolichenic bacterial counts showed higher mean values for the former (Fig 6a and 6c) than for the latter (Fig 6b and 6d) (p<0.05). Nevertheless, we cannot rule out that the lower bacterial counts within lichen thalli could be due, at least in part, to the thalli disinfection step, usually utilized in similar studies [6,15,20]. Disinfection, and other stress factors, affect bacterial viability and/or culturability, especially on solid rich media [45,47–49,56,57,61,62]. However, injured or stressed bacterial cells can recover their culturability under favorable conditions, such as exposure to host nutrients [45,48,56–58]. Furthermore, the recovery of bacteria from oligotrophic environments can be improved in media with low carbon content [63]. This could explain, to a certain extent, the fact that the culturability of endolichenic bacteria from disinfected thalli remained highest on the nutrient poor ABL medium with lichen extract (Fig 6b and 6d).

### Natamycin, extended washing without disinfection and thalli disruption by crushing further improve the recovery of lichen associated bacteria

**Fig 7:**
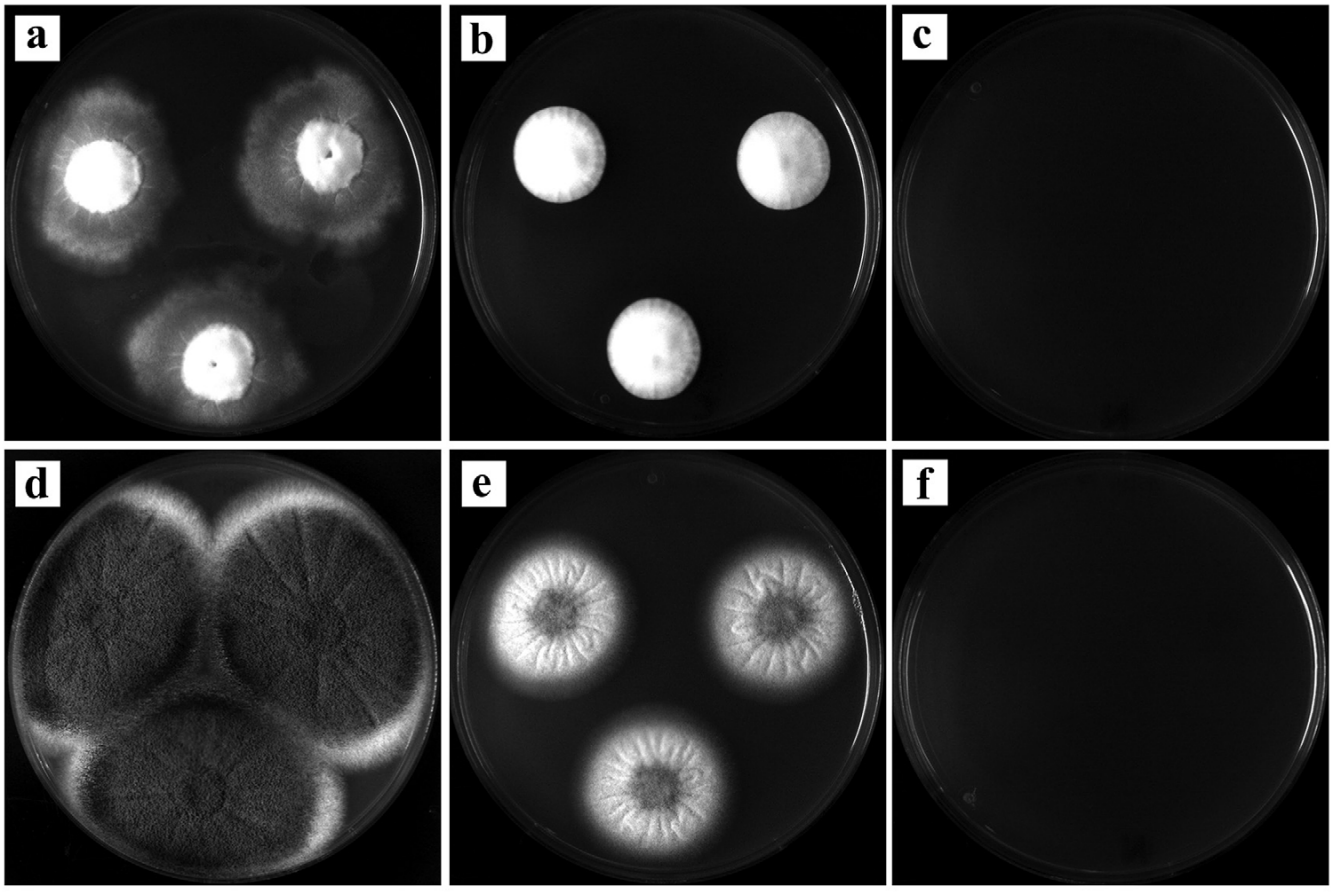
Effect of cycloheximide and natamycin on the growth of filamentous fungi from *P*. *furfuracea* samples. Control KB medium without fungicide (a,d) and representative examples of the effect of cycloheximide (b,e) and natamycin (c,f) on the growth of *Fusarium* sp. (above) and *Penicillium* sp. (below), two filamentous fungi frequently isolated from lichen thalli samples after 7 days incubation on the complex medium KB at 25°C under dark conditions.

**Fig 8:**
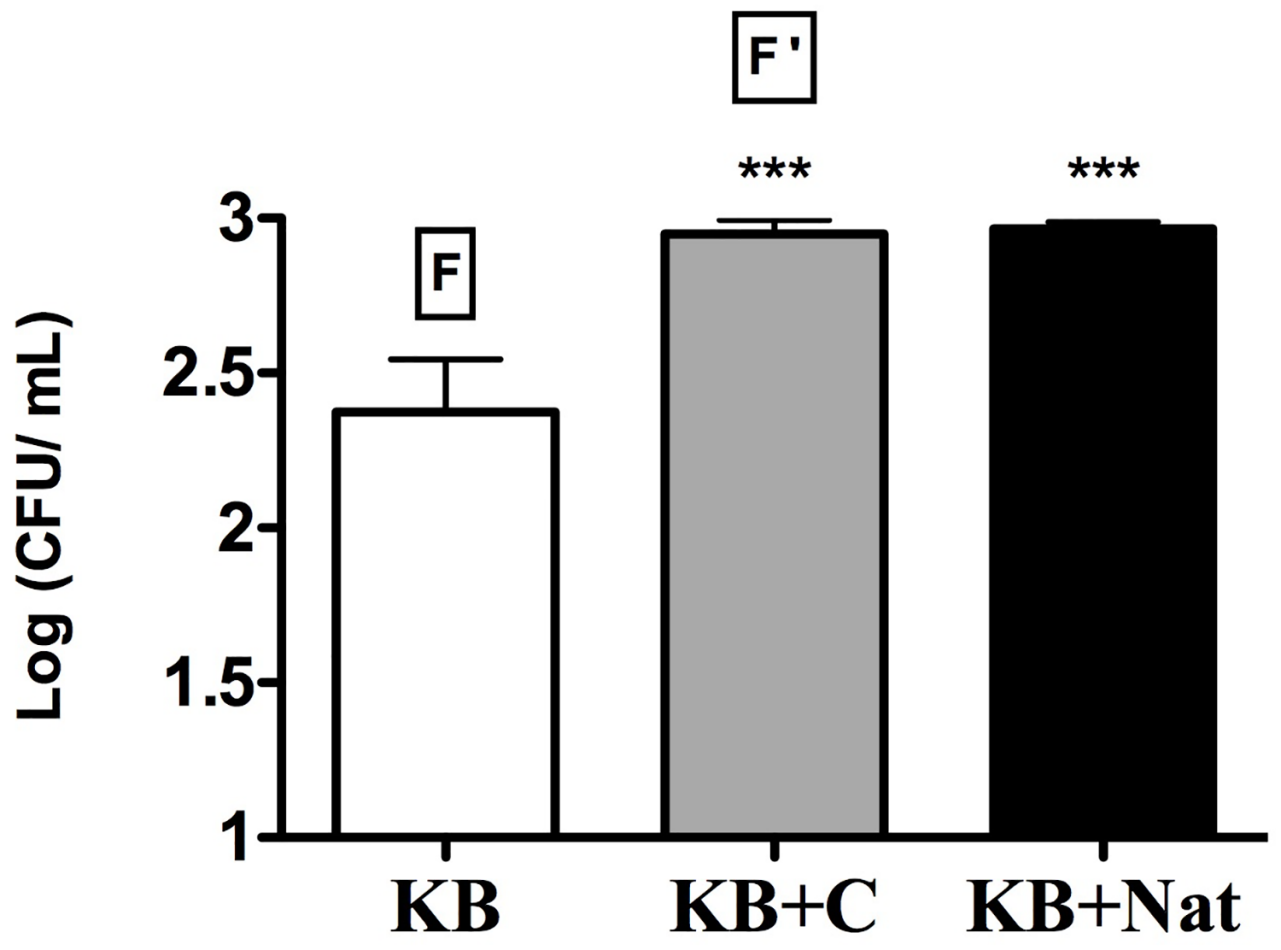
Effect of cycloheximide and natamycin on the recovery of *P*. *furfuracea* associated bacteria. Mean culturable counts of *P*. *furfuracea* ectolichenic bacteria from washed thalli (CFU/ml) on complex medium KB, supplemented with cycloheximide (C) or natamycin (Nat). F, filamentous fungi that invade plates over a period of 3–5 days. F ', filamentous fungi that invade plates after 7 days at 25°C under dark conditions. Each bar represents the mean value counts on each medium from two different *P*. *furfuracea* thalli analyzed in duplicate. Standard deviations are represented by vertical lines. Asterisks indicate statistically significant differences (*** p<0.0001) with respect to control KB medium without fungicide.

To avoid the growth of cycloheximide resistant filamentous fungi, usually appearing within a week on bacterial isolation plates, we evaluated the fungicide natamycin on a selection of seven resistant fungi often grown on KB plates. Natamycin was able to suppress the growth of the fungi tested (see representative pictures in Fig 7). In addition, when applied for the recovery of *P*. *furfuracea* associated bacteria on KB, fungal growth was delayed, reduced and/or inhibited for more than 15 days (Fig 8). Consequently, natamycin was used in subsequent bacteriological analyses.

**Fig 9:**
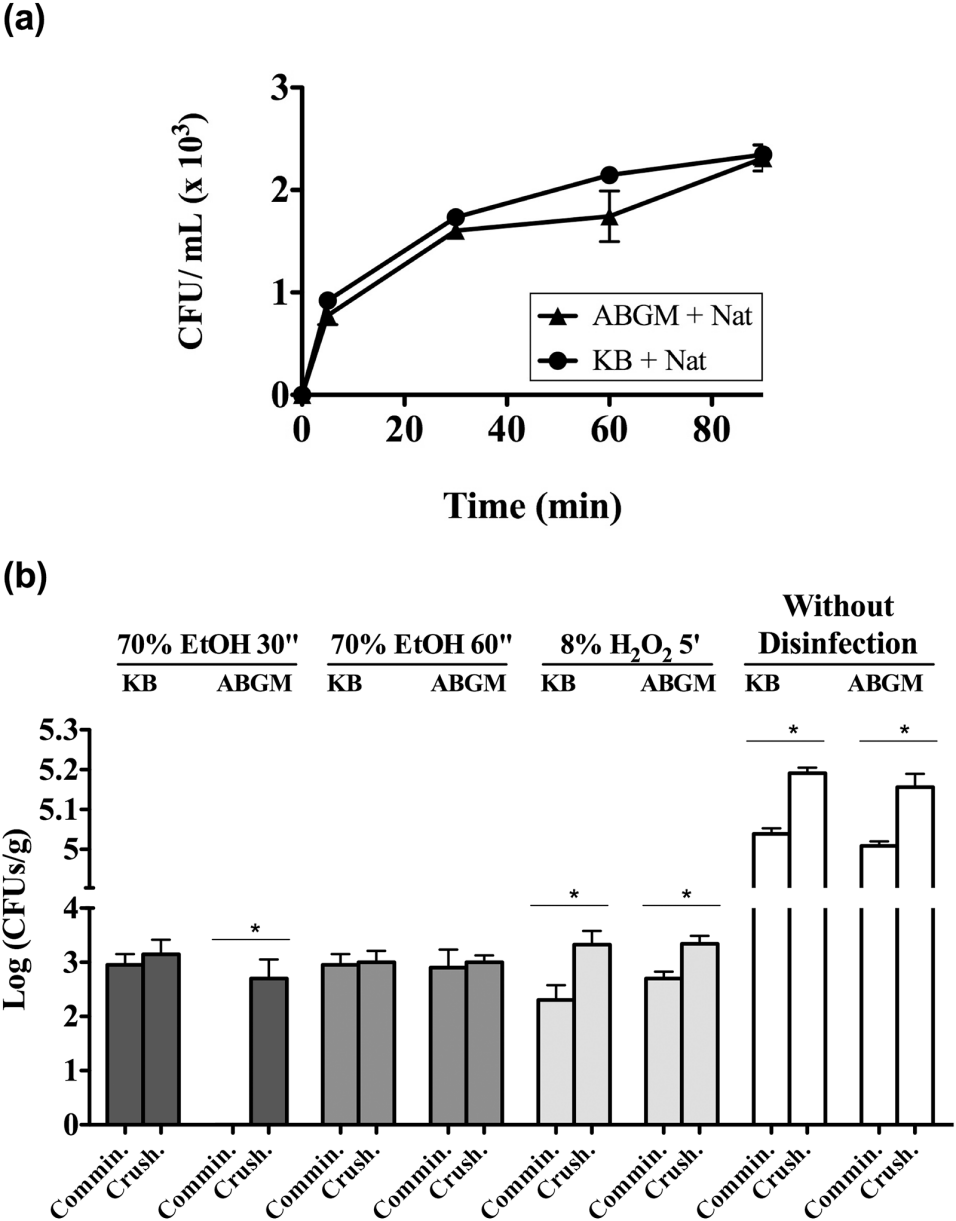
Effect of washing time and disinfection treatments and processing protocols on the recovery of bacteria from *P*. *furfuracea*. Details concerning washing time (a) and the type and/or time of disinfection tested (b) on KB and ABGM media after 7 days of incubation at 25°C under dark conditions are shown in the figure. The processing protocols (b) compared were comminution (Commin.) *versus* crushing (Crush.). Each symbol (a) or bar (b) represents the mean value of duplicate counts on natamycin-supplemented media, from the same bulk thalli sample (five thalli from different trees). Standard deviations are represented by vertical lines. Asterisks indicate statistically significant differences (* p<0.05) between comminution *versus* crushing on the two culture media used. Significant differences were also observed between disinfection *versus* non-disinfection treatments (p<0.05).

In order to ascertain whether thalli disinfection could affect the recovery of endolichenic bacteria, several disinfection procedures were compared to extended washing without disinfection. Simultaneously, we also evaluated a faster thalli disruption method. As shown in Fig 9a, mean counts of recovered ectolichenic bacteria increased significantly (p<0.05) in parallel to washing time, reaching stable values between 60 and 90 min (about 10^3^ CFU/ml) (Fig 9a). Interestingly, isolation of endolichenic bacteria from washed thalli sub-samples without disinfection led to the recovery of ca. 10^5^ CFU/g (Fig 9b). These counts were about 100 times higher (p<0.01) than their counterparts after disinfection (about 10^3^ CFU/g), showing that this step leads to an underestimation of the number, and probably the diversity of culturable endolichenic bacteria. Additionally, in most cases, mean bacterial counts were significantly higher in thalli processed by crushing (p<0.05) (Fig 9b) than in those processed by comminution. Accordingly, extended washing of thalli without disinfection and thalli disruption by crushing were selected for later analyses.

**Fig 10:**
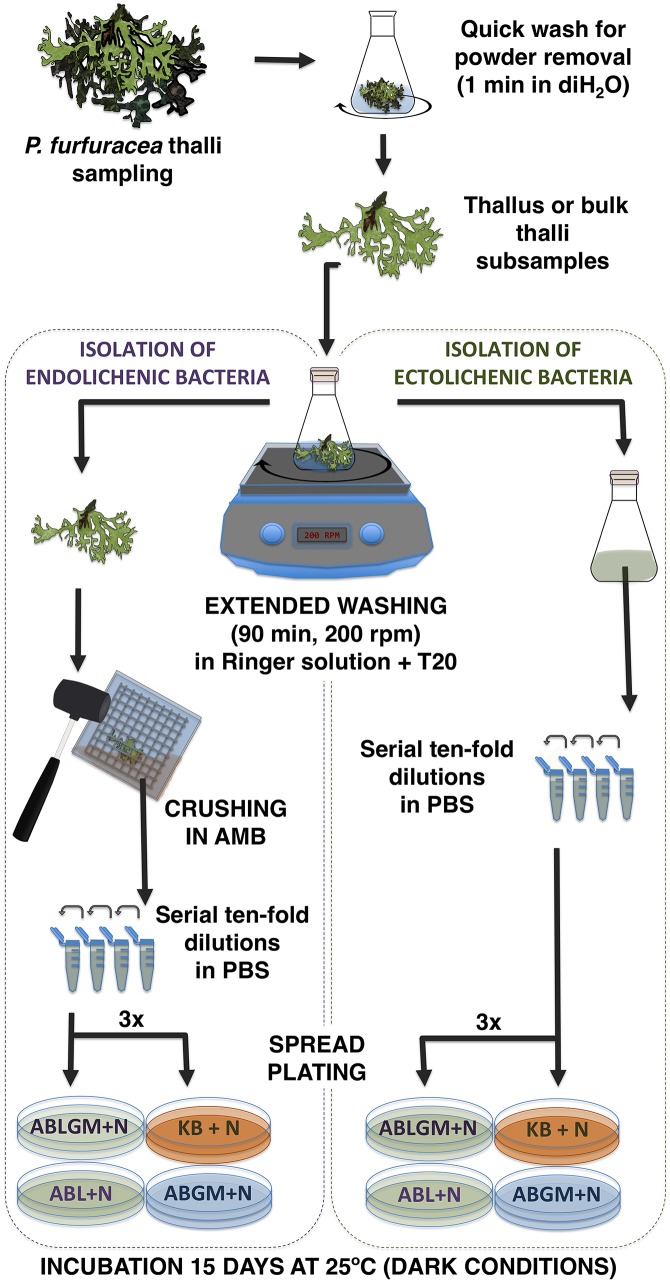
Scheme of the optimized protocol steps for bacteriological analysis of lichen samples. After collecting fresh thalli samples a quick wash is performed to remove environmental powder. Thereafter, either single or bulk thalli subsamples are processed for isolation of lichen associated bacteria. An extended washing in Ringer solution plus Tween 20 is performed to improve the recovery of bacteria from the lichen surface, and thalli washings are analyzed for ectolichenic bacteria isolation. The washed thalli are then crushed in AMB to isolate endolichenic bacteria. Ecto- and endolichenic culturable bacterial populations are estimated in triplicate on lichen enriched media with or without defined carbon sources (ABLGM and ABL), and optionally also on lichen free medium with carbon sources (ABGM) and complex rich medium KB. Spread inoculated plates are incubated at 25°C for 15 days under dark conditions.

In summary, the optimized isolation and culture approaches (Fig 10) include: i) extended washing of thalli with RST to increase the recovery of ectolichenic bacteria, thus allowing us to discard the disinfection of thalli, hence enhancing endolichenic bacteria recovery, ii) utilizing the antioxidant buffer AMB to prevent, or reduce, oxidative stress during thalli disruption by crushing, and iii) the use of lichen enriched media supplemented with the fungicide natamycin.

**Fig 11:**
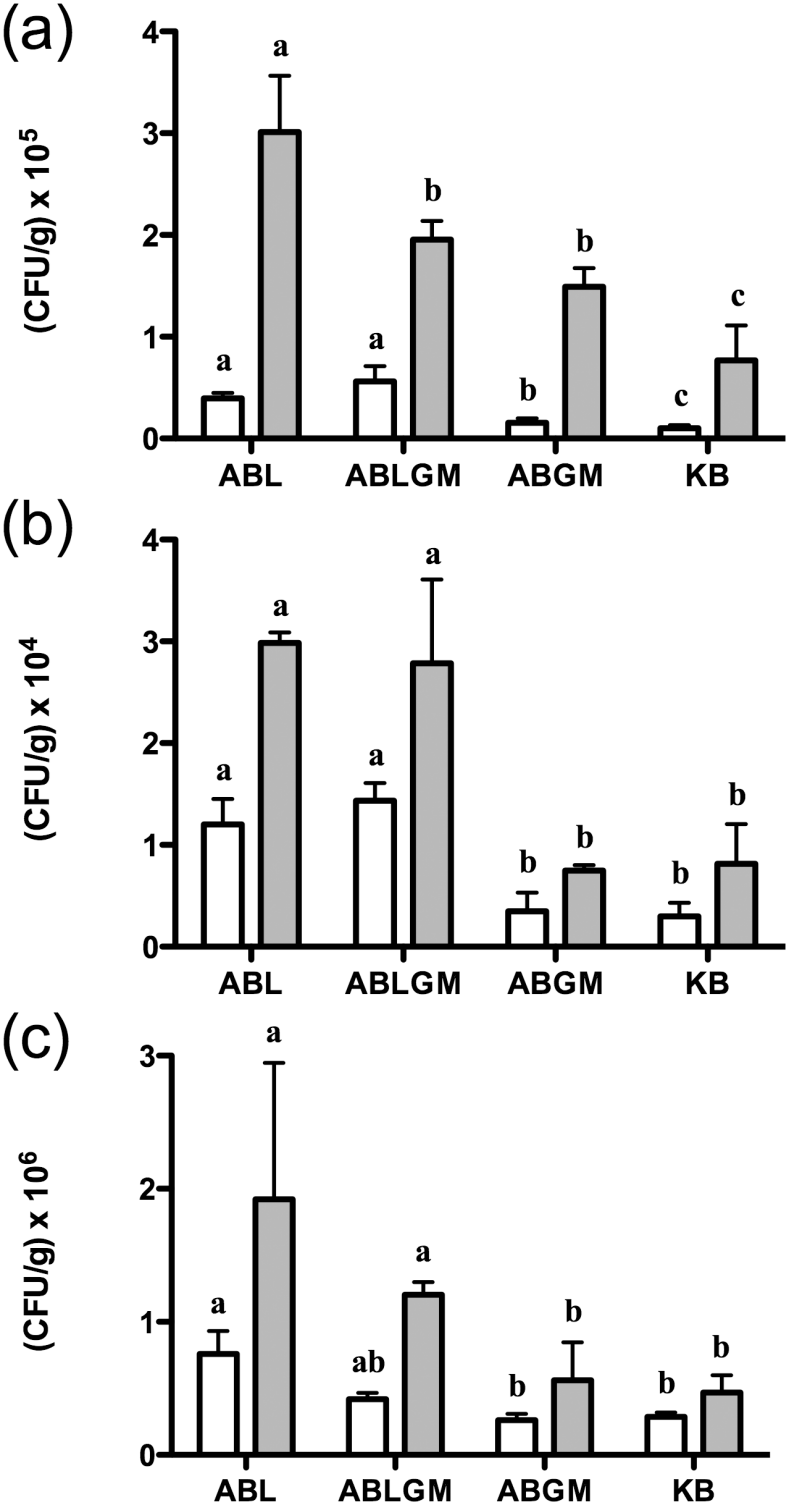
Recovery of bacteria associated with *P*. *furfuracea* (a), *R*. *farinacea* (b) and *P*. *pseudotinctorum* (c) with the optimized processing protocol and lichen enriched media. Mean culturable counts of ectolichenic bacteria from washed thalli (white bars) and endolichenic bacteria from thalli tissue (grey bars) of bulk samples (five thalli from different trees) on lichen enriched media with or without defined carbon sources (ABLGM and ABL), and also on lichen free medium with carbon sources (ABGM) and KB after 15 days of incubation at 25°C under dark conditions. Each bar represents the mean value of triplicate counts on each medium from the same bulk thalli sample. Standard deviations are indicated by vertical lines. Different letters were used to indicate statistically significant differences (p<0.05) among the different culture media utilized, for either ecto- or endolichenic bacterial counts.

When the optimized methodology was applied to new bulk samples of *P*. *furfuracea*, ecto- and endolichenic bacterial populations increased up to 10^4^ CFU/g and 10^5^ CFU/g, respectively (Fig 11a). In the case of ectolichenic bacteria, population sizes were again higher on ABL and/or ABLGM compared to lichen free media (p<0.05) (Fig 11a). However, endolichenic bacterial communities reached their highest densities on the low nutrient ABL medium, which recovered larger culturable bacteria than ABLGM, ABGM and KB (p<0.01). In this respect, Grube et al. [6] reported considerable bacterial abundances in other lichen species by using a nutrient poor medium developed for enumeration of oligotrophic water bacteria [64].

To validate the optimized innovative methodology, it was applied for the analysis of thalli samples of other lichen species (*R*. *farinacea* and *P*. *pseudotinctorum*) from different habitats and locations, obtaining similar results (Fig 11b and 11c). In *R*. *farinacea* ecto- and endolichenic bacteria counts ranged between 103–10^4^ CFU/g (Fig 11b), and in the case of *P*. *pseudotinctorum* they reached 10^5^ and 10^6^ CFU/g, respectively (Fig 11c). In general, bacterial counts were significantly higher on media enriched with *R*. *farinacea* or *P*. *pseudotinctorum* extract (ABL and ABLGM) than on lichen free media (p<0.05). However, it was not evident from these results which of the two lichen enriched media was more effective for the recovery of either ecto- or endolichenic bacteria. Accordingly, we propose the use of ABL and ABLGM media for further studies of lichen inhabiting bacteria, which would also increase the diversity of the isolated bacteria.

It is worth mentioning that in the lichen species assayed, lichen enriched media allowed the growth of bacteria unable to grow on lichen free media. The former confirms that the growth of previously uncultured lichen symbiotic bacteria can be achieved just by providing the appropriate nutrients (i.e. fresh lichen extracts of the lichen species of interest), and underlines the relevance of lichens as a promising source of novel and singular microorganisms.

Interestingly, in the populations of the different lichen species analyzed in our study (Fig 11), bacterial culturable counts from internal thalli were higher than those from the thalli surface, agreeing with previous studies by *in situ* analysis of bacterial communities associated with other lichen species [16]. This result could be related to different nutrient availability on thallus surface *versus* inner thallus tissue, as well as a lower level of protection for bacteria on lichen surfaces against abiotic stresses, such as UV radiation or lower water availability, as well as meteorological events. Related to this, Cardinale et al. [17] using a microscopic approach reported that the age, the type of substrate-inhabited and sun exposure affect the abundance of bacterial cells in lichen thalli.

Overall, the present work constitutes the first study focused on the development of a standardized methodology to improve the recovery of bacteria associated with lichens. Furthermore, we provide, for the first time, data on the abundance of culturable ecto- and endolichenic bacteria that naturally colonize the lichen species *P*. *furfuracea*, *R*. *farinacea* and *P*. *pseudotinctorum*, some of which only grew on lichen enriched media and could be considered lichen specific symbionts. The innovative methodology developed is also applicable to other microorganisms inhabiting these and other lichen species. In the future, it will significantly increase our knowledge of multiple species of symbionts associated with lichens, which is essential for understanding microbial interactions in the functioning of lichen symbiosis. It may also prove useful for the isolation of novel and unique microorganisms with diverse biotechnological potential as yet to be discovered.

## Supporting Information

S1 Fig Boxplot representation of lichen enriched media effect on the number of colony types of *P*. *furfuracea* associated bacteria from bulk thalli samples. Data are from two different samplings (a,b and c,d) with three replicates each. Data shows colony numbers of ectolichenic bacteria from washed thallus (a,c) and endolichenic bacteria from thallus tissue (b,d) on lichen enriched media (LEM) *versus* lichen free media (LFM) after 15 days of incubation at 25°C under dark conditions. A significantly higher number of colony types was recorded on the lichen enriched media compared to the lichen free media in b, c and d (p<0.05). Circles represent outliers in the data.
(TIF)
