# Supplementary figures and images for: Innovative Approaches Using Lichen Enriched Media to Improve Isolation and Culturability of Lichen Associated Bacteria
Source: PLoS One. 2016 Aug 5;11(8):e0160328. doi: 10.1371/journal.pone.0160328 (PMC4975499; doi:10.1371/journal.pone.0160328)

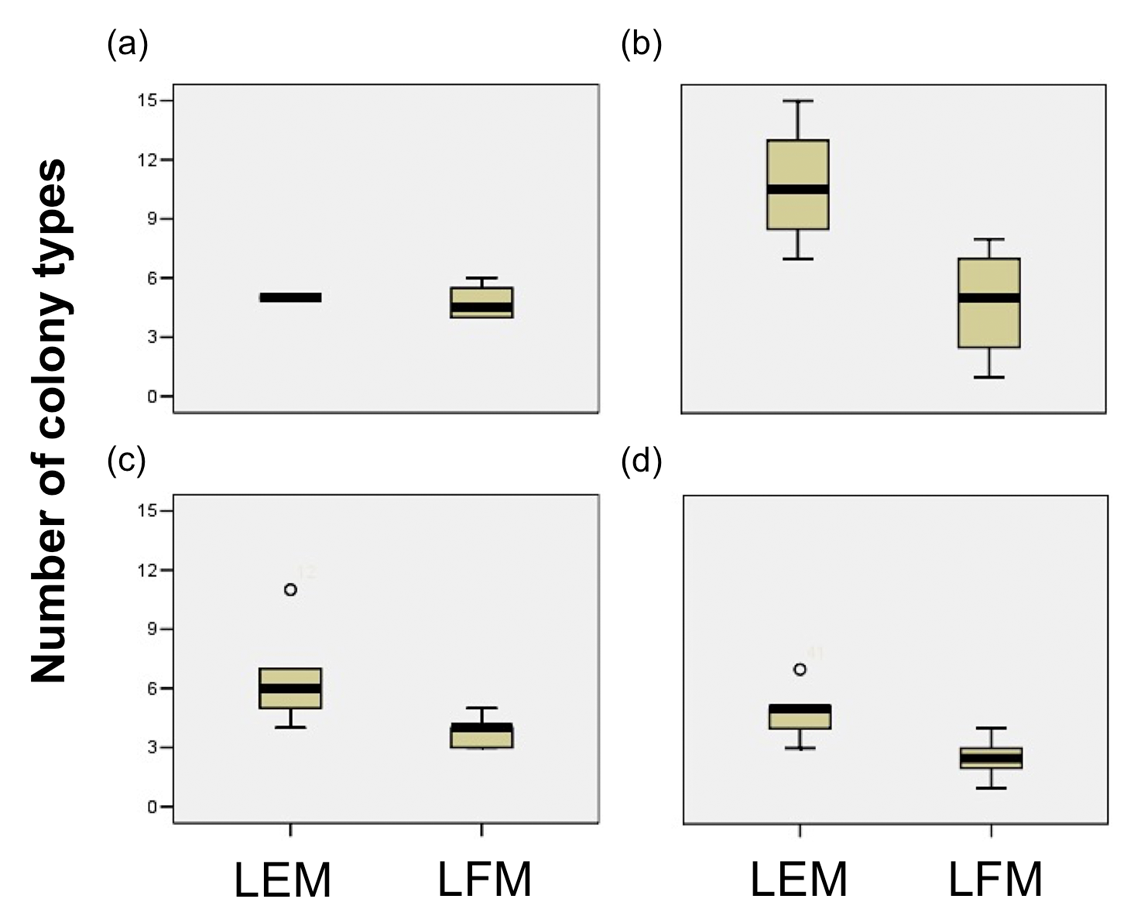

Supplement: S1 Fig — Data are from two different samplings (a,b and c,d) with three replicates each. Data shows colony numbers of ectolichenic bacteria from washed thallus (a,c) and endolichenic bacteria from thallus tissue (b,d) on lichen enriched media (LEM) versus lichen free media (LFM) after 15 days of incubation at 25°C under dark conditions. A significantly higher number of colony types was recorded on the lichen enriched media compared to the lichen free media in b, c and d (p<0.05). Circles represent outliers in the data. (TIF) [file pone.0160328.s001.tif]
